# Exercise Training and Interventions for Coronary Artery Disease

**DOI:** 10.3390/jcdd9050131

**Published:** 2022-04-25

**Authors:** Hugo Fernández-Rubio, Ricardo Becerro-de-Bengoa-Vallejo, David Rodríguez-Sanz, César Calvo-Lobo, Davinia Vicente-Campos, José López Chicharro

**Affiliations:** 1 Faculty of Nursing, Physical therapy and Podiatry, Universidad Complutense de Madrid, 28040 Madrid, Spain; hugofern@ucm.es (H.F.-R.); ribebeva@enf.ucm.es (R.B.-d.-B.-V.); davidrodriguezsanz@ucm.es (D.R.-S.); 2 Faculty of Health Sciences, Universidad Francisco de Vitoria, Pozuelo de Alarcón, 28223 Madrid, Spain; davinia.vicente@ufv.es; 3 Grupo FEBIO, Universidad Complutense de Madrid, 28040 Madrid, Spain; jlopezch@ucm.es

**Keywords:** coronary artery disease, resistance training, respiratory muscle training, aerobic training, exercise

## Abstract

Coronary artery disease (CAD) may be considered a main cause of mortality and the prevalence of CAD is increasing nowadays, leading to high health costs in many countries. Despite the fact of the regression of the atherosclerotic plaque, the decrease in blood viscosity and the growth of collateral vessels have been proposed as improvements that CAD patients may obtain under exercise performance. Thus, the present narrative review aimed to carry out a brief specific analysis of the results achieved when performing endurance, strength or inspiratory muscle training. Exercise attenuates certain pathophysiological processes of this disease, such as endothelial dysfunction or the vulnerability of atherosclerotic plaques, and produces improvements in functional capacity and muscle strength, among others. Within the different exercise modalities, the most important parameter to be considered seems to be the total caloric expenditure, and not so much the modality itself. As such, in cardiac rehabilitation, when prescribing exercise, we should possibly focus on the modality that obtains more adherence in patients. To conclude, it must be highlighted that total caloric expenditure is not being taken into account when comparing interventions and this relevant information should be considered in future studies.

## 1. Introduction

Worldwide, cardiovascular disease may be considered a main cause of mortality, with 40% of this mortality rate attributable to coronary artery disease (CAD) [1,2]. In addition, the prevalence of CAD is increasing nowadays, leading to high health costs in many countries and being the main cause of morbidity and disability in developed countries [3,4]. Some risk factors for this disease are chronic blood pressure elevation, hypercholesterolemia, diabetes, age, obesity, smoking and physical inactivity, considering this lack of physical activity as an independent predictor for the development of this pathology [5,6,7,8,9]. The risk factors for CAD involve some pathological processes in the coronary arteries, such as endothelial dysfunction, macrophage infiltration in the intima, and lipid influx into the artery, forming foam cells in early atherosclerotic lesions [2,8]. As the size of the lesion increases, a thickening of the neointima occurs due to the fact that the coronary smooth muscle cells (CSM) migrate towards the intima, increasing the synthesis of macromolecules of the extracellular matrix [10]. Further, the phenotypic modulation of the CSM will present a more osteogenic phenotype that will lead to vascular calcification [10], and the macrophages and CSM will undergo apoptosis and accumulate in the centre of the atherosclerotic plaque, forming a lipid-laden necrotic nucleus [10]. Such vulnerable and unstable plaques are characterized by having a large lipid-laden nucleus with a thin, fibrous cap; they also contain cholesterol crystals and will be prone to rupture and thrombosis [2]. Finally, this atherosclerotic plaque will produce stenosis in the coronary vessels limiting the blood flow in them, and manifesting angina and/or myocardial infarction [2,8,11,12].

Likewise, patients with CAD will present low-grade chronic systemic inflammation that will affect the vascular system [13]. Vascular inflammation involves the activation of platelets and leukocytes, which is an early characteristic of atherosclerotic disease since platelets will produce inflammatory and mitogenic molecules (interleukins, chemokines…) that facilitate the adhesion of monocytes and leukocytes to the endothelium. After transmigrating from the endothelial layer, these cells differentiate into foam cells and promote the inflammatory process of atherosclerotic plaques [14].

Endothelial dysfunction in damaged blood vessels leads to decreased nitric oxide (NO) concentration in vascular smooth muscle cells, which has been recognized as a determinant condition for atherogenesis [15,16,17,18,19,20]. Intact endothelium is essential to protect the vessel from damaging factors and serves as a crucial modulator of vasomotor tone [21]. Moreover, it will be essential for the release of circulating progenitor cells (CPC) by the bone marrow. Among the functions of these cells, the repair of damaged endothelium, improvement of neoangiogenesis and reduction of the formation of neointima after a vascular injury have been described [8,14]. Therefore, endothelial dysfunction plays a key role in the pathogenesis of exercise-induced angina pectoris, which will lead to increased smooth muscle contraction in the vessel wall at the time of catecholamine release during exercise and decreased CPC function [1,14,18]. At the same time, these patients suffer from a decrease in the coronary vasodilator reserve, since they maintain chronic vasodilation of the coronary arteries to compensate for the stenosis produced by the atherosclerotic plaque, and if the coronary output is increased there will be difficulties to increase coronary blood flow [22].

Scientific evidence explains changes that occur in the modulation of the autonomic nervous system (ANS) after a coronary event due to an increase in the activity of the sympathetic system and a decrease in the parasympathetic system [23]. This increased sympathetic activity decreases cardiomyocyte viability, increases heart rate, decreases diastolic filling time, increases oxygen demand, and reduces coronary perfusion time. Finally, this imbalance may present deleterious effects, increasing the risk of sudden cardiac death [2,24]. Consequently, this ANS imbalance that occurs in patients with CAD severely alters heart rate variability (HRV) and heart rate recovery (HRR). Specifically, the reduction of these two parameters is a strong predictor of mortality risk, sudden cardiac death or cardiac arrhythmias [24,25]. It should be noted that HRV is a parameter related to the vagus nerve; just as the HRR is also related to the activation of the vagus nerve, since the recovery following the interruption of the exercise occurs due to a parasympathetic reactivation and a sympathetic deactivation [25,26,27,28,29,30].

In addition, a progressive loss of skeletal muscle mass since the reduction in cardiac output and tissue hypoxia in CAD induce the expression of myostatin and inflammatory cytokines [31,32,33].

Due to the pathophysiological processes suffered by patients with CAD, they are susceptible to receiving health treatment both to mitigate the symptoms of the disease and to carry out secondary prevention. There are various therapeutic approaches, such as pharmacological interventions (statins, beta-blockers…), surgical treatments (bypass and coronary stent) and cardiac rehabilitation (CR), which may be highlighted. In this review, we will develop the current evidence on cardiac rehabilitation.

Cardiac rehabilitation is a comprehensive intervention consisting of exercise, risk factor education, behaviour change and psychological support that address common risk factors for coronary artery disease (CAD) [33]. In particular, exercise therapy is recognized as an integral component of cardiac rehabilitation, and the American Heart Association (AHA) guideline [34] recommends aerobic training (AT) and resistance training (RT) as the core elements of exercise-based cardiac rehabilitation [1,5,35]. Furthermore, exercises in CR have been shown to be a safe and well-established intervention [35]. Exercise is the most important stimulus to increase myocardial oxygen demand [22,36]. The heart does not present a remarkable capacity to increase oxygen absorption, so the increase in demand must be achieved by increasing coronary blood flow (CBF), and, consequently, the coronary arteries play a relevant role [1]. This exercise-generated demand to repeatedly increase CBF results in improved myocardial compensatory oxygen supply and increased aerobic exercise capacity [5,22,35,37]. This is important since exercise capacity (EC) is a strong and independent predictor of cardiovascular and all-cause mortality in cardiovascular disease [4,38]. It has been previously shown that an increase in one metabolic equivalent (MET) in CE is associated with a 12–16% improvement in survival [39,40], and due to this, exercise results in a therapeutic intervention to be taken into account for the primary and secondary treatment of CAD [2,22].

In the meta-analysis carried out by Dibben et al. [41], which is the most recently published Cochrane review, conducted a comprehensive analysis of how CR affected all causes of mortality, such as cardiovascular mortality, myocardial infarction, coronary artery bypass graft (CABG) revascularization, or percutaneous coronary intervention (PCI), hospitalizations for all causes, hospitalizations for cardiovascular events and quality of life in addition to performing a cost-benefit assessment compared to a control group that did not exercise. One factor to highlight in this study is the division of the results into short-term (6 to 12 months), medium-term (>12 to 36 months) and long-term (>36 months). The short-term results were that exercise-based CR probably leads to a small reduction in all-cause mortality, a large reduction in MI, and a large reduction in all-cause hospitalization. Exercise-based CR probably results in little or no difference in the risk of cardiovascular mortality, CABG, and PCI up to a 12-month follow-up. Current evidence is uncertain about the effects of exercise-based CR on cardiovascular hospitalization. At medium-term follow-up, although there may be little or no difference from all causes, MI, PCI, CABG, and all-cause hospitalization, a large reduction in cardiovascular mortality was found. The evidence is uncertain for the difference in risk of cardiovascular hospitalization. With long-term data, although there may be little or no difference in all-cause mortality, exercise-based CR may lead to a large reduction in cardiovascular mortality and myocardial infarction. The evidence is uncertain for CABG and PCI. There was evidence that exercise-based CR may slightly increase the quality of life on several subscales up to 12 months of follow-up; however, these may not be clinically important differences. All eight trial-based economic evaluation studies showed exercise-based CR to be a potentially cost-effective use of resources in terms of gains in quality-adjusted life-years [41].

After this brief introduction to exercise in CR in patients with CAD, we are going to focus on how exercise may improve certain pathological processes explained above.

## 2. Bibliographic Search

The database used to perform this review was Pubmed and the keywords used for the bibliographic search were “exercise”, “coronary heart disease”, “artery coronary disease”, “resistance training”, “aerobic training”, “inspiratory muscle training” and “physical activity”.

## 3. Effects of Exercise in CAD Patients

The contribution of NO to endothelial function is significant due to its important vasodilatory and vasoprotective properties that help prevent the pathological changes that occur during CAD. Specifically, NO protects the vascular wall by inhibiting platelet aggregation, adhesion molecule expression and leukocyte penetration into the arterial intima and media layers, and lipid oxidation. These effects of NO may attenuate atherosclerosis by protecting the vessel from injury and from fatty lesion generation [2,18,42]. In this sense, investigations were carried out on whether exercise could increase NO concentrations in patients with CAD since they present a decrease in its concentration. Data obtained concluded that exercise increases the production and bioavailability of NO: (1) increasing the quantity and activity of the endothelial enzyme NOS (eNOS), (2) increasing the availability of the substrate eNOS (L-arginine) and essential cofactors (tetrahydrobiopterin (BH 4)), and (3) decreasing NO degradation by reactive oxygen species (ROS) [1,16,18,19,43]. Shear stress is one of the main physiological activators of eNOS mRNA and protein, providing a key link between NO production and exercise, as the latter involves repeated episodes of increased shear stress [1,16,18,19,43]. Likewise, the shear stress generated by exercise increases the activity of the enzyme superoxide dismutase (SOD) and hemo-oxigenase-1 (HO-1) which, being antioxidant enzymes, decrease the generation of ROS. There also seems to be a feedback mechanism between the endothelial extracellular SOD and eNOS, as NO was found to enhance extracellular SOD expression [14,18]. Another factor that decreases the amount of ROS is physical training since it inhibits ROS-generating enzymes such as nicotinamide adenine dinucleotide phosphate oxidase (NADPH) [18].

This increase in NO seems to be the main cause of the increase in coronary blood flow velocity reserve (CFVR) in CAD patients after CR since the increase in NO causes an increase in cGMP and this, in turn, can cause vasodilation of the coronary arteries when the demands for oxygen supply increase. Likewise, this improvement in CFVR is due to the fact that the increase in NO leads to a partial recovery of the endothelial function which in turn causes an increase in the tone of the coronary artery; therefore, they present a greater vasodilator capacity [1,2].

It should be noted that the increase in NO together with the improvement in endothelial function in patients with CAD who performed physical training causes an increase in CPC levels [8,14,18,21,44]. These cells promote vasculogenesis and the myocardial expression of vascular growth factors (inducing the remodelling of pre-existing capillaries and arterioles) [8,14].

Another vasoprotective and antiatherosclerosis effect of regular exercise training in CAD patients is due to the reduction in platelet reactivity and the number of platelet-leukocyte conjugates, although further randomized controlled trials would be necessary to determine the clinical impact of these findings [14].

Furthermore, a very interesting benefit of CR in these patients is that the atherosclerotic lesion changes from a vulnerable atherosclerotic plaque to a more stable plaque, making it less prone to rupture and consequently could reduce cardiac mortality [45]. This stabilization of the atherosclerotic plaque in the groups that performed exercise occurs for various reasons, such as the conversion of the osteogenic phenotype of CSM to a contractile and healthy one, a higher content of collagen and elastin and a lower concentration of macrophages [2,10,42].

On the other hand, two meta-analyses [13,46] investigated the effect of exercise on the inflammatory activity of these patients. They observed that exercise could induce an anti-inflammatory effect. Specifically, Thompson et al. [13] showed that there was evidence of how exercise produced significant beneficial effects on the C-reactive protein, fibrinogen and von Willebrand factor, but no significant differences were detected in interleukin-6 (IL6), interleukin-8 (IL8), interleukin-10 (IL10), tumor necrosis factor-alpha (TNFα), vascular cell adhesion molecule (VCAM), intercellular adhesion molecule-1 (ICAM1), E-Selectin, P-Selectin and normal T-cell expressed and secreted (RANTES). This reduction in inflammatory activity may be due to the fact that these patients have elevated basal levels, or to an improvement in the main risk factors for CAD that promote inflammation, such as obesity, diabetes, hypertension and dyslipidaemia [13].

**Figure 1:**
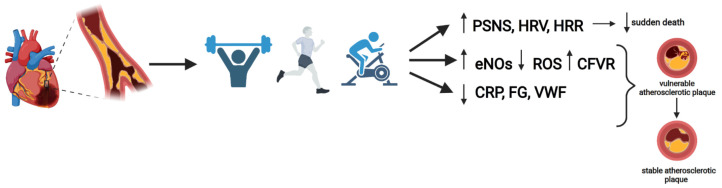
Beneficial effects of exercise training on the CAD risk factors including inflammation, sympathetic activity and endothelial dysfunction. Created with BioRender.com accessed on 1 March 2022. CAD: Coronary artery disease; PSNS: parasympathetic nervous system; HRV: heart rate variability; HRR: heart rate recovery; CFVR: coronary blood flow velocity reserve; CRP: C-reactive protein; VWF: Von Willebrand Factor; FG: fibrinogen.

To observe the effects of physical training on the ANS, Manresa-Rocamora et al. [25] carried out a meta-analysis in order to study the possible changes that exercise could exert on the modulation of the ANS in these patients, for which they analysed heart rate variability (HRV), HRV index calculated by the square root of the differences in the successive RR interval (RMSSD) and heart rate recovery (HRR). The results indicated that HRV at rest did not reveal statistically significant differences between groups. Nevertheless, significant differences were found to improve both HRR and RMSSD in the exercise-based CR group. In addition, the authors stated that current evidence seems to suggest that RMSSD might be better suited than HF to reflect resting PNS function because RMSSD is less affected by respiratory influences and methodological limitations [25]. Finally, exercise-based CR improves parasympathetic function in CAD patients, but at rest, it is difficult to draw a concise conclusion due to discrepancies between HRV and RMSSD [25]. Figure 1 shows the beneficial effects of exercise training on the CAD risk factors including inflammation, sympathetic activity and endothelial dysfunction.

Despite the fact of the regression of the atherosclerotic plaque, the decrease in blood viscosity and the growth of collateral vessels have been proposed as improvements that CAD patients may obtain under exercise performance, although there is still no clear evidence to confirm these changes are so significant to produce a benefit [14,42]. After carrying out an analysis of the improvements obtained in general when performing different types of exercise in relation to the pathophysiological processes of CAD, we are going to carry out a brief specific analysis of the results achieved when performing endurance, strength or inspiratory muscle training.

## 4. Aerobic Training (AT)

The meta-analyses carried out by Chen et al. [4] and Kraal et al. [47] analysed the effects of aerobic training in CAD patients on peak VO_2_ (Table 1). In both studies, a statistically significant improvement (*p* < 0.05) was obtained in this parameter compared to the control group. In addition, Chen et al. [4] studied the effects of aerobic exercise on systolic and diastolic blood pressure, LDL-C and HDL-C concentrations, triglycerides, total cholesterol levels and left ventricular ejection fraction (LVEF). There was an improvement in all of them when compared to the control group except for the diastolic blood pressure and triglycerides and total cholesterol levels. In this way, the present results show evidence of how aerobic exercise has positive effects on some modifiable cardiovascular risk factors such as LDL-C, HDL-C and systolic blood pressure, also showing an improvement in the functional capacity of patients with improvement of peak VO_2_ and LVEF. On the other hand, these effects of exercise may be decisive, since a 1% reduction in LDL-C reduces the risk of coronary events by approximately 2% and for each 1% decrease in the LVEF value, the probability of death increases by a factor of 1.04 [4].

The meta-analysis carried out by Gomes-Neto et al. [35] analysed which aerobic training intervention was more effective in increasing peak VO_2_ in these patients, comparing high-intensity interval training (HIIT) or moderate-intensity continuous training. In a first analysis, they reached a significant difference in peak VO_2_ for patients in the HIIT group compared to those in continuous training, however, when energy expenditure was equal between both interventions (isocaloric exercises), there was a non-significant difference in peak VO_2_ between both modalities of training. These results were previously evidenced by Vromen et al. [48] when performing a meta-regression analysis on different aerobic exercise modalities in patients with chronic heart failure. It was concluded that total energy expenditure seemed to be the only characteristic of training with a significant effect on the improvement of exercise capacity exercise. This was also shown in the study performed by Kraal et al. [47] observing that the total energy expenditure was significantly associated with improvement in exercise capacity showing that an increase in energy expenditure of 100 J·kg^−1^ was related to an improvement in peak VO_2_ of 0.91 mL·min^−1^·kg^−1^.

In this way, with the current evidence, the aerobic training modality is not as important as the total energy expenditure of the session, so the aerobic training modality that produces greater adherence to the patient should be prioritized to achieve determined energy expenditure. Caution should be noted, as excessive exercise/calorie expenditure may hinder improvement in exercise capacity [47].

## 5. Resistance Training

In this section, we will present the results of five meta-analyses [33,49,50,51,52] with a different structure when comparing the groups. In the study performed by Hollings et al. and the study carried out by Fan et al. [50,52], a resistance training group (RT) was studied against a control group that did not exercise (CG), RT versus an aerobic training group (AT) and finally the combination of RT and AT (CT) versus AT. In the study performed by Xanthos et al. [49], CT versus AT, RT versus AT, and also CT versus RT were analysed. The meta-analysis carried out by Marzolini et al. [51] only compared CT versus AT. Finally, the article performed by Yamamoto et al. [33] compiled two types of randomized controlled trials, some comparing RT versus usual care and others CT vs. AT. In their meta-analysis, they combined both types of trials so that the intervention group was RT and CT, and the control group was AT and usual care. Likewise, the meta-analysis by Yamamoto et al. [33] performed an analysis by age groups, grouping it into middle-aged patients (<65 years) and older patients (>65 years). After this brief introduction of the articles, in the following paragraphs, we will present the results obtained (Table 2).

In the meta-analysis performed by Yamamoto et al. [33], both intervention groups (middle-aged and older) showed statistically significant differences in favour of the control group in upper and lower extremity strength and functional capacity by increasing both peak VO_2_ and time of exercise. Nevertheless, in the middle-aged intervention group, significant differences were not achieved in mobility compared to the control group, and in the older intervention group, significant improvements were obtained compared to the control group.

In the meta-analysis carried out by Xanthos et al. [49], CT compared to AT presented a significant effect in favour of CT for peak VO_2_, maximal work capacity, and muscle strength. In RT compared to AT for peak VO_2_ and muscle strength, no significant evidence was shown to suggest an effect in favour of RT or AT. Finally, for CT versus RT, only the study performed by Vona et al. [53] observed that both peak VO_2_ and maximum work capacity presented a significantly greater effect in favour of the CT group.

In the meta-analysis carried out by Marzonlini et al. [51], when comparing CT versus AT for peak VO_2_ there were no significant differences between both interventions despite the existence of a trend in favour of CT. Nevertheless, significant differences were shown in favour of CT in maximal exercise capacity, ventilatory threshold, fat-free mass, percentage of body fat, and upper and lower body muscle strength.

In the meta-analysis carried out by Fan et al. [52] regarding the CT versus AT comparison, significant differences were found in favour of CT in peak VO_2_, quality of life in the physical and global component, upper and lower body muscle strength, anaerobic threshold and LVEF; in contrast, no significant differences were found between the two interventions in maximal VO_2_, the emotional component of quality of life, and the left ventricular end-diastolic dimension (LVEDD). When comparing RT versus AT for peak VO_2_ and quality of life, no significant difference was found between groups, however, regarding anaerobic threshold and LVEF, the authors only found one study in which significant differences were observed in favour of RT. Finally, in the analysis of RT compared to CG for peak VO_2_, quality of life, LVEF and LVEDD, there were significant differences in favour of RT.

In the meta-analysis performed by Hollings et al. [50] regarding the CT versus AT comparison for peak VO_2_, no significantly greater difference was found between both interventions, but a significant difference was found in favour of CT for maximum work capacity and muscle strength of the upper and lower body. In the analysis of RT versus AT, no significant differences were observed between peak VO_2_ and work capacity, and for muscle strength, the authors only found two studies and did not have enough data to pool them. Finally, in the comparison of RT versus CT in terms of muscle strength of the upper and lower body, significant differences were found in favour of RT, in the analysis of peak VO_2_ and work capacity, the authors stated that the heterogeneity was significant, so it was not suitable for grouping.

Of note, current evidence suggests that RT or CT have similar adverse events as aerobic training alone [49].

It can be noted that the intervention of RT in patients with CAD is an effective measure to increase exercise capacity and muscle strength. Increased muscle strength is interesting as it is associated with a better prognosis and functional performance, promoting independent living and return to work after a cardiac event. On the other hand, impaired muscle strength predicts a higher mortality rate in patients with CAD. Considering that RT is the most powerful exercise modality for improving strength, its inclusion in CR programs is very important [33,50].

To conclude this section, it is necessary to highlight that, in futures studies, when making comparisons of CT versus AT or RT, the total caloric expenditure should be considered to equate it and show with certainty whether the benefits of CT are due to the combination of RT and AT or only because it facilitates a greater total energy expenditure.

## 6. Inspiratory Muscle Training

Inspiratory muscle weakness is defined as a reduced maximum inspiratory pressure (MIP), either in its absolute value (70–80 cm H_2_O) or in relation to its normal value (<70% of the normal value). Importantly, this weakness is associated with mobility problems, an increased risk of myocardial infarction, and higher rates of all-cause and CVD mortality in CAD patients. In addition, the weakness of this musculature is related to reductions in lung function contributing to dyspnea on exertion. This weakness presents a higher prevalence in patients with CAD compared to healthy controls; it should be noted that these patients who underwent CABG surgery presented a reduction of 17–36% in MIP from the preoperative to the postoperative state, and this is associated with greater pulmonary complications in the postoperative period [54].

Due to the benefits of inspiratory muscle training (IMT) in patients with heart failure [55,56,57,58,59] and the weakness of the inspiratory muscles that these patients usually present, our research group decided to study the possible effects of IMT. We found 10 randomized clinical trials [60,61,62,63,64,65,66,67,68,69] and one quasi-experimental study [70] (Table 3). All these studies were carried out in patients with CAD who underwent surgery for coronary artery bypass grafting (CABG) or were scheduled to receive this intervention; the two studies [71,72] of patients with CAD who were not going to undergo surgery were discarded due to the study performed by Darnley et al., which did not present a control group, and the study carried out by Muammer et al. was exclusively in patients with metabolic syndrome. Eight of the studies selected compared IMT versus usual care [61,62,63,64,65,66,67,69], two of them analysed CT plus IMT versus CT [60,70] and one study compared AT + IMT versus AT [68].

The respiratory muscle’s function was evaluated in all these studies, although with differences between them. The MIP was evaluated in all these studies and a significant difference was shown in favour of the IMT group with respect to the control group, except in the study provided by Matheus et al. [69] in which no significant difference was observed. It is worth mentioning that the IMT intervention in this study was only 3 days post-surgery. Inspiratory muscle resistance (SMIP) was analysed in three studies [60,62,67], two of these studies showed significant differences in favour of IMT [62,67]. Maximum expiratory pressure (MEP) was analysed in five studies [63,66,68,69,70], and two of them showed significant differences in favour of IMT [63,70].

Functional capacity was studied through the parameters of peak VO_2_ or the 6-min walk test (6MWT). Peak VO_2_ was analysed in four studies [60,63,68,70]. The only one that could not show significant differences in favour of IMT was the study performed by Miozzo et al. [68]. The 6MWT was evaluated in four studies [60,63,66,68]. Regarding the peak VO2, Miozzo et al. [68] did not find significant evidence in favour of the IMT compared to the control group.

Lung capacity was studied in five studies [61,63,66,67,69] through various parameters. The forced vital capacity (FVC) and the maximum expiratory volume in the first second (FEV1) were studied in four articles [61,63,66,67], and only significant differences in favour of the IMT were found in the study performed by Stein et al. [63] for both parameters. The relationship between FEV1 and FVC (FEV1/FVC) was evaluated in the study carried out by Savci et al. [66] detailing no significant differences between groups. The vital capacity was analyzed in two studies [61,69] and only the study performed by Matheus et al. [69] found significant differences in favour of IMT. Tidal volume and maximum expiratory flow were evaluated in the study performed by Bertolini et al. [69], showing significant differences in favour of IMT compared to the control group for tidal volume, but these differences were not observed for the peak expiratory flow.

Post-operative pulmonary complications were analysed in three studies [61,62,65], two of them analysed pneumonia [62,65] and another one atelectasis [61], with all of them showing significant differences in favour of IMT compared to the control group.

Alveolar-arterial oxygen gradient and oxygen saturation were studied by Turky et al. [64], detailing for both parameters a significant difference between groups in favour of IMT.

The length of hospital stay was evaluated by four studies [61,62,65,66]. In all these studies, a significant difference was observed in favour of the IMT group, with the exception of the study performed by Hulzebos et al. [61]. Furthermore, Savci et al. [66] analysed the duration of intensive care after surgery, detailing significant differences in favour of IMT.

General muscle strength was only studied by Miozzo et al. [68] reporting any significant differences between groups. On the other hand, the study carried out by Dos Santos et al. [60] was the only one that analysed the effects of IMT on four blood biomarkers, such as endothelial function (NOx), inflammatory profile (C-reactive protein), oxidant profile (AOPP) and antioxidant profile (FRAP), reporting significant differences in favour of IMT compared to the control group in FRAP.

Quality of life was analysed in five studies [60,65,66,68,70] using different questionnaires. Two studies [60,70] used the Minnesota Living with Heart Failure Questionnaire (MLHFQ) and both of them showed a significant difference in favour of IMT compared to the control group. Two studies used the SF-36 questionnaire [65,68] and significant differences were not found between both groups. The study performed by Valkenet et al. [65] also used the EQ-5D-3L and, as for the SF-36, significant differences were not found. Lastly, Savci et al. [66] evaluated the quality of life with the Nottingham Health Profile (NHP) and the Hospital Anxiety and Depression Scale (HADS). For the NHP, a significant difference in favour of the IMT was found in the dimension of sleep, but not for the remaining five dimensions, such as emotional reactions, social isolation, energy, pain and physical mobility. Significant differences were shown for HADS in favour of IMT for anxiety, but not for depression.

With these results, it seems that IMT results in a useful therapy for patients with CAD before or after CABG surgery, since it improves the function of the inspiratory muscles, the functional capacity, decreases hospital stay and decreases postoperative lung complications.

These results must be taken with caution due to the great heterogeneity between the studies. This is due to the fact that the intervention protocols in the studies are very varied with respect to the intensity of the IMT or the duration of the intervention. In addition, the collection of measurements was very varied over time with respect to the surgical intervention.

To conclude, we believe that it may be of great health interest to carry out IMT investigations in patients with CAD who are not going to undergo surgery and who adopt a conservative treatment.

## 7. Exercise Prescription

The prescription of exercise may be considered one of the most important interventions linked to cardiac rehabilitation or cardiovascular disease prevention. The benefits of exercise are generally well known, but the dosage of exercise to obtain benefits is not so well known. Therefore, current evidence regarding the prescription of aerobic training (AT) and resistance training (RT) was detailed for both patients with CAD and for the prevention of this disease.

Aerobic exercise prescription needs to define the appropriate intensity for training in both primary and secondary prevention. For healthy subjects, the appropriate exercise intensity is usually based on the percentage of maximum heart rate reserve (MHRR) or percentage of maximum oxygen consumption reserve (VO_2_R) [73].

Nevertheless, it is advisable to accurately adjust the exercise intensity in patients with CAD by performing a stress test until a number of abnormal signs or symptoms are reproduced and thus the appropriate intensity should be set at least 10 bpm below the HR. Signs and symptoms are the appearance of angina or other symptoms of cardiovascular failure, plateau or decrease in systolic blood pressure, systolic blood pressure > 240 mm Hg or DBP > 110 mm Hg, ST depression of 1 mm, horizontal or descending, increased frequency of ventricular arrhythmias, other significant electrocardiogram alterations, or other signs or symptoms of exercise intolerance [73,74].

It should be noted that in approximately 35% of the medical centers where cardiac rehabilitation is carried out, this stress test is performed to prescribe exercise intensity. If this test is not available, the two parameters used to find an adequate intensity are the RPE scale (6–20) or resting HR plus 20 bpm (for patients with myocardial infarction, CABG or patients using β-blockers) or plus 30 bpm (for patients not using β-blockers). On the RPE scale, values of 11 to 13 are recommended for early exercise sessions and values of 12 to 15 for future training sessions [73,74].

Considering all these data in order to calculate the appropriate intensity, duration and frequency of continuous aerobic training for healthy subjects and for patients with CAD were specified in Table 4 [73,74,75,76]. It should be noted that in patients with CAD, a progression of aerobic training should be performed, starting with the minimum thresholds reflected in Table 4. When the exercise progression is performed, the frequency and duration should be increased, and finally, the intensity (RPE) should be also increased [73].

For the prescription of strength training in patients with CAD, loads from 40% to 50% of one repetition maximum (RM) are recommenced and should include one to two sets from 12 to 15 repetitions of exercises working the major muscle groups at a frequency from two to three times per week. In addition, isometric contractions of maximum intensity should be avoided in these patients, as these contractions can cause a drastic increase in blood pressure. In healthy subjects, in the strength prescription, the recommended loads vary from 60% to 80% of one RM and should include from two to three sets of 8–12 repetitions of exercises working the main muscle groups with a frequency of at least 2 days a week (Table 5) [73,75,76].

The initiation of an RT program in patients with CAD is usually contraindicated if they present unstable angina, uncontrolled arrhythmias, left ventricular outflow obstruction, symptomatic heart failure, severe valvular disease, and uncontrolled hypertension. In addition, it would also be advisable for the left ventricular ejection fraction to be greater than 35% and a functional capacity ≥5 METs before starting this program [73].

Finally, current evidence suggests that exercise modality or other training variables (e.g., intensity) is less important than total weekly caloric expenditure. Currently, a caloric expenditure greater than 1500 kcal per week seems to be sufficient to delay the progression of the disease. Therefore, when prescribing exercise, apart from taking into account all the aforementioned variables, it would be very important to assess the patient’s preferences in order to try to produce the greatest possible adherence to the program, and therefore a greater total caloric expenditure [47,48,73].

## 8. Conclusions

There is currently enough evidence to affirm that exercise is an effective therapy for the primary and secondary prevention of CAD. Exercise attenuates certain pathophysiological processes of this disease, such as endothelial dysfunction or the vulnerability of atherosclerotic plaques, and produces improvements in functional capacity and muscle strength, among others.

Within the different exercise modalities, the most important parameter to be considered seems to be the total caloric expenditure, and not so much the modality itself. Thus, in cardiac rehabilitation, when prescribing exercise, we should possibly focus on which is the modality that obtains more adherence in patients.

To conclude, it must be highlighted that in most of the studies, total caloric expenditure is not being taken into account when comparing interventions and we believe that it is a relevant piece of information to consider in future studies.

## Figures and Tables

**Table 1:** Effects of aerobic training in patients with artery coronary disease.

Studies	Groups	Parameters Improving Significantly (*p* < 0.05) with Aerobic Training	Parameters Not Improving Significantly (*p* < 0.05) with Aerobic Training
Chen, 2017	AT vs. CG	Peak VO_2_, LDL-C, HDL-C, LVEF and SBP	DBP, triglycerides and total cholesterol levels
Kraal, 2017	AT vs. CG	Peak VO_2_	-

AT: aerobic training; CG: control group; DBP: diastolic blood pressure; LVEF: left ventricular ejection fraction; SBP: systolic blood pressure.

**Table 2:** Effects of resistance training in patients with coronary artery disease.

Studies	Groups	Parameters Improving Significantly (*p* < 0.05) with Resistance Training	Parameters Not Improving Significantly (*p* < 0.05) with Resistance Training
Yamamoto, 2016	(Older) RCT vs. AT	Peak VO_2_, lower extremity strength, time of exercise and mobility	-
	(Middle-aged) RCT vs. AT	Peak VO_2_, lower extremity strength and time of exercise	Mobility
Fan, 2021	RT vs. CG	Peak VO_2_, quality of life, LVEF and LVEDD	-
	RT vs. AT	Anaerobic threshold and LVEF	Peak VO_2_ and quality of life
	CT vs. AT	Peak VO_2_, quality of life in the physical and global component, upper and lower body muscle strength, anaerobic threshold and LVEF	Maximal VO_2_, the emotional component of quality of life, and LVEDD
Hollings, 2017	RT vs. CG	Upper and lower body muscle strength	-
	RT vs. AT	-	Peak VO_2_ and work capacity
	CT vs. AT	Maximal work capacity and upper and lower body muscle strength	Peak VO_2_
Xanthos, 2017	RT vs. AT	-	Peak VO_2_ and muscle strength
	CT vs. AT	Peak VO_2_, maximal work capacity and muscle strength	-
Marzolini, 2012	CT vs. AT	Maximal exercise capacity, ventilatory threshold, fat-free mass and upper and lower body muscle strength	Peak VO_2_

AT: aerobic training; CG: control group; CT: combination of RT and AT; LVEDD: the left ventricular end-diastolic dimension; LVEF: left ventricular ejection fraction; RCT: resistance training or combined training; RT: resistance training.

**Table 3:** Effects of inspiratory muscle training in patients with coronary artery disease.

Studies	Groups	Parameters Improving Significantly (*p* < 0.05) with Inspiratory Muscle Training	Parameters Not Improving Significantly (*p* < 0.05) with Inspiratory Training
Dos Santos, 2019	IMT vs. CG	MIP, peak VO_2_, 6MWT, FRAP and MLHFQ	SMIP, CRP, NOx and AOPP
Hulzebos, 2006 [61]	IMT vs. CG	MIP and post-operative pulmonary complications	FVC, FEV1, VC and length of hospital stay
Hulzebos, 2006 [62]	IMT vs. CG	MIP, SMIP, post-operative pulmonary complications and length of hospital stay	-
Stein, 2009	IMT vs. CG	MIP, MEP, peak VO_2_, 6MWT, FVC and FEV1	-
Turky, 2017	IMT vs. CG	MIP, alveolar-arterial oxygen gradient and oxygen saturation	-
Valkenet, 2017	IMT vs. CG	MIP, post-operative pulmonary complications and length of hospital stay	SF-36 questionnaire and EQ-5D-3L
Savci, 2011	IMT vs. CG	MIP, 6MWT, length of hospital stay, duration in intensive care, NHP (sleep) and HADS (anxiety)	FVC, FEV1, FEV1/FVC, NHP (emotional reactions, pain, energy, social isolation and physical mobility) and HADS (depression)
Weiner, 1998	IMT vs. CG	MIP and SMIP	FVC and FEV1
Miozzo, 2018	AT + IMT vs. AT	MIP	MEP, peak VO_2_, 6MWT, muscle strength and SF-36 questionnaire
Matheus, 2012	IMT vs. CG	VC and TV	MIP, MEP and MEF
Hermes, 2015	IMT	MIP, MEP, peak VO_2_ and MLHFQ	-

6MWT: 6-min walk test; AOPP: oxidant profile; AT: aerobic training; CG: control group; CRP: C-reactive protein; FEV1: maximum expiratory volume in the first second; FRAP: antioxidant profile; FVC: forced vital capacity; HADS: Hospital Anxiety and Depression Scale; IMT: inspiratory muscle training; MEF: maximum expiratory flow; MEP: maximum expiratory pressure; MIP: maximum inspiratory pressure; MLHFQ: Minnesota Living with Heart Failure Questionnaire; NHP: Nottingham Health Profile; NOx: endothelial function; SMIP: inspiratory muscle resistance; VC: vital capacity; TV: tidal volume.

**Table 4:** Prescription of Aerobic training.

Variable	Recommendation, CAD Patient	Recommendation, Healthy Subject
Mode	Continuous aerobic training	
Frequency	Three to five sessions per week	
Duration	20–60 min per session	
Intensity	- 10 bpm below the HR associated with any signs or symptoms - Resting HR plus 20 bpm or 30 bpm - RPE 11–13 (first sessions) - RPE 12–15 (next sessions)	- 55%/65% to 90% maximal HR - 40%/50% to 85% maximal VO_2_R

bpm: beats per minute; CAD: coronary artery disease; HR: Heart rate; VO_2_R: Maximum Reserve Oxygen Consumption; RPE: Rating of perceived exertion.

**Table 5:** Prescription of resistance training.

Variable	Recommendation, CAD Patient	Recommendation, Healthy Subject
Frequency	Two or three times per week	Two times per week
Sets/Repetitions	1–2 sets/12–15 repetitions	2–3 sets/8–12 repetitions
Loads	40–50% of the 1 RM	60–80% of the 1 RM

CAD: coronary artery disease; RM: maximum resistance.

## Data Availability

Data will be available upon request to the corresponding author.
